# *Aspergillus oryzae* fermentation modifies the composition of Massa Medicata Fermentata and its effects on the gut microbiota in mice with spleen-deficiency constipation

**DOI:** 10.3389/fmicb.2026.1897277

**Published:** 2026-08-19

**Authors:** Xianqing Sun, Qin He, Liwen Li, Zhoujin Tan, Xuejuan Liang

**Affiliations:** 1 Hunan Academy of Chinese Medicine, Changsha, Hunan, China; 2 School of Traditional Chinese Medicine, Hunan University of Chinese Medicine, Changsha, Hunan, China

**Keywords:** 16S rRNA sequencing, *Aspergillus oryzae*, fungal fermentation, gut microbiota, Massa Medicata Fermentata, short-chain fatty acids, spleen-deficiency constipation

## Abstract

This study investigated the effects of *Aspergillus oryzae* fermentation on the chemical composition of Massa Medicata Fermentata (MMF) and the gut microbiota in mice with spleen-deficiency constipation by directly comparing unfermented MMF (UFL) with *A. oryzae*-fermented MMF (AOFL). We examined whether *A. oryzae* fermentation altered the small-molecule chemical profile of MMF and whether, compared with UFL, AOFL was associated with more pronounced changes in selected host functional indices and gut microbial profiles. The model was induced using *Folium Sennae* decoction, restricted water access, and a low-fiber diet, after which mice received UFL or AOFL. Untargeted LC–MS was used to characterize putatively annotated chemical features in the two preparations. Fecal water content, serum D-xylose, motility-related serum markers, hepatic oxidative-stress indices, colonic histology, concentrations of short-chain fatty acids (SCFAs) in colonic contents, and 16S rRNA-based microbial profiles were assessed in parallel. Fermentation with *A. oryzae* altered the putatively annotated chemical profile of MMF; L-phenylalanine and hypoxanthine showed significantly higher relative signal intensities in AOFL, whereas the other selected features showed nonsignificant directional differences. Relative to UFL, AOFL showed higher serum D-xylose, 5-hydroxytryptamine, and motilin levels, greater hepatic superoxide dismutase activity, and longer colonic crypts. Relative to the model group, AOFL showed lower malondialdehyde and vasoactive intestinal peptide levels and higher gastrin levels and goblet cell number. Microbiota analysis suggested that AOFL partially shifted the disturbed community structure toward the normal-control pattern and altered predicted metabolic functions. None of the measured SCFAs differed significantly between the AOFL and model groups; acetate and propionate were numerically higher with AOFL, whereas butyrate showed no corresponding recovery. Overall, *A. oryzae* fermentation altered the putatively annotated chemical composition of MMF and was associated with a partial shift in gut microbial composition and more pronounced changes in selected host functional indices than UFL.

## 1 Introduction

Constipation is a common functional gastrointestinal disorder in clinical practice. It is mainly characterized by reduced defecation frequency, difficult defecation, and dry stools, which markedly impair quality of life and increase the risk of related conditions, including hemorrhoids, anal fissure, abdominal distension, and abdominal pain (Suares and Ford, 2011; Bharucha and Lacy, 2020). Current treatments for constipation largely include bulk-forming laxatives, osmotic laxatives, stimulant laxatives, and prokinetic agents. Although these therapies can relieve symptoms, long-term use may still be associated with diarrhea, abdominal pain, electrolyte disturbances, and variable therapeutic responses (Chang et al., 2023). Therefore, safe interventions with multi-target regulatory potential are needed for chronic constipation, particularly when it is interpreted within traditional Chinese medicine patterns. In TCM, although the primary site of constipation is considered to be the large intestine, its occurrence is closely related to the transportation and transformation functions of the spleen and stomach. Spleen deficiency impairs transportation and transformation, weakens the propulsion of qi, and disrupts the distribution of body fluids, thereby impairing large intestinal conduction and leading to dry stools and difficult evacuation. Constipation with spleen-deficiency syndrome, hereafter referred to as spleen-deficiency constipation, is often accompanied by fatigue, poor appetite, abdominal distension, shortness of breath, reduced speech/low verbal activity, or weak defecatory force. Its prevention and treatment should therefore focus on regulating the spleen and stomach, strengthening spleen qi, and restoring intestinal conduction function.

From a modern microecological perspective, the manifestations attributed to spleen deficiency may overlap with impaired digestion and absorption, altered nutrient utilization, and disturbed gastrointestinal functional regulation (Zhang et al., 2022). These changes may modify the quantity and composition of substrates entering the colon and alter the intestinal physicochemical environment, thereby contributing to gut microbial dysbiosis and abnormal microbial metabolism (You et al., 2022; Chen et al., 2025; Lv et al., 2026; Zheng et al., 2026). In turn, an altered gut microbiota may influence intestinal function through microbial metabolites, neuroendocrine signaling, mucus-barrier homeostasis, immune regulation, and oxidative-stress-related processes. Disturbances in these interconnected pathways may be associated with reduced intestinal secretion, impaired motility-related regulation, mucosal injury, and the development of dry stools and difficult defecation (Rendeli et al., 2023; Zheng et al., 2025; Jiang et al., 2026). Thus, gut microbiota and its metabolites provide a potential modern biological framework for interpreting the relationship between spleen-deficiency-related dysfunction and constipation, although the two conceptual systems should not be regarded as directly equivalent.

Consistent with this microecological framework, increasing evidence indicates that gut microbiota participates in the development and progression of constipation. Gut microbiota contributes to the regulation of intestinal function by influencing the production of short-chain fatty acids (SCFAs), intestinal neuroendocrine signaling, the mucus barrier, local immune responses, and oxidative stress status (Portincasa et al., 2022; Liu et al., 2025). In constipation, the diversity and composition of gut microbiota are often altered, accompanied by abnormal levels of microbial metabolites (Fan et al., 2022; Tuohongerbieke et al., 2024). Acetate, propionate, and butyrate are the major SCFAs produced by gut microbial fermentation of dietary fiber and undigested carbohydrates (Koh et al., 2016). Although acetate, propionate, and butyrate are all SCFAs, they differ in their producing microorganisms, host utilization patterns, and biological functions (Koh et al., 2016; Ríos-Covián et al., 2016). Therefore, integrated analysis of gut microbial structure, the SCFA profile, and host gastrointestinal function may help explain the effects of traditional Chinese medicine on spleen-deficiency constipation from the perspective of modern microecology.

Massa Medicata Fermentata (MMF) is a traditional fermented Chinese medicine that is used to strengthen the spleen, harmonize the stomach, promote digestion, and resolve food stagnation. It is commonly used for patterns such as food retention, epigastric and abdominal distension, and impaired transportation and transformation of the spleen and stomach (Chinese Pharmacopoeia Commission, 2020). Unlike conventional Chinese herbal pieces, the functional properties of MMF depend not only on the composition of the original medicinal materials but also on the microbial transformation of starch, proteins, polysaccharides, and plant-derived components during fermentation (Fan et al., 2024). Fermentation can alter the small-molecule composition of medicinal materials, including sugars, organic acids, amino acids, nucleosides, and volatile compounds, thereby affecting their intestinal transformation, utilization, and microecological effects. *A. oryzae* is a commonly used fermenting microorganism in traditional fermentation systems. It has a strong capacity to secrete extracellular enzymes, including amylases, proteases, and glycosidases, which promote substrate degradation and metabolite accumulation (Chancharoonpong et al., 2012; Daba et al., 2021; Sun et al., 2024). Therefore, *A. oryzae* fermentation may affect the intestinal transformation and utilization of MMF by changing its putative compound composition, and may further regulate gut microbial structure and microbial fermentation metabolism.

Recent studies on Massa Medicata Fermentata and related fermented Chinese medicines have begun to link processing-dependent chemical changes with intestinal microecological effects. Comparative studies have shown that different fermentation methods, formulation compositions, and post-fermentation processing can produce distinct chemical profiles and gut microbiota-related responses (Fan et al., 2024; Wang et al., 2024). In spleen-deficiency constipation models, different MMF formulations have also been associated with changes in intestinal enzyme activities, serum D-xylose and motility-related markers, and microbial composition (Liang et al., 2025). These findings suggest that fermentation conditions and formulation composition may jointly shape the chemical characteristics and intestinal microecological effects of fermented medicinal masses.

However, direct comparisons between MMF fermented with a defined *A. oryzae* inoculum and a composition-matched unfermented preparation remain limited, particularly in the context of spleen-deficiency constipation. Compared with earlier MMF studies centered on preparation standardization and quality evaluation, the present study focuses on the biological consequences of a defined-strain fermentation intervention through an integrated framework combining LC–MS-based untargeted chemical profiling, SCFA quantification, 16S rRNA sequencing, and a multi-indicator assessment of host gastrointestinal function. Accordingly, the present study compared unfermented MMF (UFL) with *A. oryzae*-fermented MMF (AOFL) to evaluate the effects of defined fungal fermentation on the putatively annotated chemical composition of MMF, gut microbial composition, and selected host-response indices in mice with spleen-deficiency constipation. Based on this framework, we formulated two hypotheses. First, fermentation with a defined *A. oryzae* inoculum would alter the small-molecule chemical profile of MMF. Second, compared with UFL, AOFL would be associated with distinct changes in gut microbial composition and more pronounced changes in intestinal absorptive-function indices, motility-related serum markers, oxidative-stress status, and colonic mucosal structure in mice with spleen-deficiency constipation.

## 2 Materials and methods

### 2.1 Experimental materials, reagents, and instruments

#### 2.1.1 Experimental animals and housing conditions

Forty specific pathogen-free male Kunming (KM) mice weighing 20 ± 2 g were purchased from Hunan SJA Laboratory Animal Co., Ltd. [license No. SCXK (Xiang) 2021-0002]. The animals were housed in the barrier facility of the Laboratory Animal Center of Hunan University of Chinese Medicine [license No. SYXK (Xiang) 2024-0014], where the room temperature and relative humidity were maintained at 23–25 °C and 50%–70%, respectively. Standard laboratory chow was provided by Beijing Huafukang Bioscience Co., Ltd. [feed license No. Jing Si Zheng (2024) 06076]. Except during the constipation-modeling period, all mice had free access to food and water. The experimental protocol was approved by the Animal Ethics Committee of Hunan University of Chinese Medicine (No. HNUCM21-2511-26; 28 May 2025), and the animal procedures were conducted in accordance with institutional requirements for laboratory-animal welfare.

#### 2.1.2 Preparation of unfermented and *A. oryzae*-fermented MMF

The MMF formula was composed of flour, wheat bran, Armeniacae Semen Amarum, Vignae Semen, Artemisiae Annuae Herba, Herba Xanthii, and Herba Polygoni Hydropiperis at a ratio of 50:50:45:45:10:10:10. Armeniacae Semen Amarum and Vignae Semen (450 g each) were ground, passed through a 60-mesh sieve, and mixed with flour and wheat bran (500 g each) as the base powder. Artemisiae Annuae Herba, Herba Xanthii, and Herba Polygoni Hydropiperis (100 g each) were decocted for 1 h with water at five times the total mass of these three herbal materials; the filtrate was concentrated to 500 g and cooled to 50 °C. UFL and AOFL were prepared by Henan Hongbo Pharmaceutical Co., Ltd. according to the procedures summarized in Table 1 and a previously reported preparation process (Liang et al., 2025). For AOFL preparation, the fermentation process was controlled using a defined *A. oryzae* inoculum amount, a temperature of 30–35 °C, and a duration of 24–48 h. The operational fermentation endpoint was defined as continuous visible white-mycelial coverage of the exposed substrate surface. After drying, both products were pulverized and passed through a 24-mesh sieve. For oral administration, UFL and AOFL powders were separately suspended in purified water at 0.2 g/mL and thoroughly mixed before gavage. A total daily dose of 4 g/kg crude-drug equivalent, divided into two administrations, was selected based on previous mouse studies using the same dose of Massa Medicata Fermentata (Wang et al., 2024; Liang et al., 2025). Medicinal materials were purchased from Henan Hongbo Pharmaceutical Co., Ltd.; *A. oryzae* powder (Cat# 0856; Shandong HezhongKangyuan Biotechnology Co., Ltd.) was used for the fermentation step.

**Table 1:** Preparation of unfermented and *A. oryzae*-fermented MMF.

Type	Preparation method
Unfermented MMF (UFL)	The herbal extract (250 g) was mixed thoroughly with 950 g of base medicinal powder. The mixture was cut into cubes directly and dried without a fermentation step.
*A. oryzae*-fermented MMF (AOFL)	*A. oryzae* powder (Cat# 0856; 3 g) was blended with 950 g of the base medicinal powder by geometric dilution. After 250 g of the concentrated herbal extract was added, the mixture was fermented at 30–35 °C for 24–48 h. Fermentation was terminated when the exposed substrate surface was continuously covered by visible white mycelia, after which the material was cut into cubes and dried.

#### 2.1.3 Preparation of *Folium Sennae* decoction

*Folium Sennae* (120 g; Anhui Jinren Chinese Herbal Pieces Co., Ltd.; batch No. 2407032) was pre-soaked in water for 30 min. After soaking, the material was decocted for 30 min with water at five times the mass of the dry herbal material and filtered through gauze. The remaining residue was decocted for a further 15 min with the same amount of water. After the two extracts were pooled, the combined decoction was concentrated to 1 g/mL using a rotary evaporator at 60 °C and 15 r/min. The final concentrated decoction was stored at 4 °C before use.

#### 2.1.4 Assay kits

MDA (Cat# BC6415) and SOD (Cat# BC5165) assay kits were purchased from Beijing Solarbio Science & Technology Co., Ltd. ELISA kits for mouse vasoactive intestinal peptide (VIP), motilin (MTL), 5-hydroxytryptamine (5-HT), D-xylose, and gastrin (GAS) were supplied by Jiangsu Jingmei Biotechnology Co., Ltd., with catalog numbers JM-02729M1, JM-02775M2, JM-02726M1, JM-12962M1, and JM-03140M2, respectively.

#### 2.1.5 Experimental instruments

The main instruments were a KZ-III-FP tissue grinder (Wuhan Servicebio Technology Co., Ltd.), a 5810R refrigerated benchtop centrifuge (Eppendorf, Germany), an LC-RE-301 rotary evaporator (Shanghai Lichen Bangxi Instrument Technology Co., Ltd.), an ELGA ultrapure water system, a SPARK multimode microplate reader (Tecan, Sweden), an R550 small-animal inhalation anesthesia system (RWD Life Science Co., Ltd., Shenzhen, China), and a JA2003 electronic balance (Shanghai Sunny Hengping Scientific Instrument Co., Ltd.).

### 2.2 Methods

#### 2.2.1 Establishment of the spleen-deficiency constipation model, grouping, and intervention

After a 3-d acclimatization period, the 40 KM mice were randomly allocated using a random-number table into four prespecified groups: the normal control group (NC), model group (MD), unfermented MMF group (UFL), and *A. oryzae*-fermented MMF group (AOFL), with 10 mice per group. Baseline body weight was recorded after group allocation and before model induction. To induce the spleen-deficiency state, mice in the MD, UFL, and AOFL groups received *Folium Sennae* decoction by gavage at 20 g/(kg·d), divided into two daily administrations, for 7 d, while food and water remained freely available. NC mice received the same volume of sterile water. From day 8, *Folium Sennae* administration was discontinued, and constipation was induced in the MD, UFL, and AOFL groups by limiting water access to 0.5 h once daily and feeding raw rice as a low-fiber diet at 4–8 g per mouse per day for 8 d. NC mice continued to receive standard chow and water ad libitum (Sun et al., 2026b).

After model establishment, the modeling procedures were discontinued, and all mice were provided with standard chow and unrestricted access to water. Mice in the UFL and AOFL groups received unfermented MMF or *A. oryzae*-fermented MMF, respectively, at a total daily dose of 4 g/kg crude-drug equivalent, divided into two gavage administrations. NC and MD mice received sterile water at the same volume and frequency for 7 d. After the intervention, terminal blood collection was performed under isoflurane anesthesia, followed by euthanasia and collection of liver, colon tissue, and colonic-content samples for subsequent analyses. Because the administered preparations were distinguishable, investigators performing gavage were not blinded. All biological samples and histological sections were coded, and investigators responsible for laboratory assays, histological assessment, sequencing-data processing, and statistical analysis were blinded to group allocation until the analyses were completed.

#### 2.2.2 Criteria for model evaluation

Model establishment was evaluated by comparing the prespecified NC and MD groups. Based on previously reported criteria (Sun et al., 2026b), the spleen-deficiency constipation model was operationally considered established when persistent spleen-deficiency-like manifestations, including curled or arched-back posture, dull fur, somnolence, reduced spontaneous activity, suppressed body-weight gain, and limb weakness, were accompanied by a qualitatively observed reduction in defecation frequency, smaller and apparently drier or firmer fecal pellets, and significantly lower fecal water content in the MD group than in the NC group.

#### 2.2.3 LC–MS-based untargeted chemical characterization of MMF samples

Dried UFL and AOFL were subjected to untargeted LC–MS analysis using three biological replicate samples per group (*n* = 3), with each sample processed and analyzed separately. Peak picking, alignment, and integration generated a matrix containing retention time, accurate mass, relative peak area, and database-matching information. Candidate features were annotated mainly from accurate mass, MS/MS fragmentation, and matches in mzCloud, mzVault, ChemSpider, MassList, and related databases. Relative peak areas were used to calculate AOFL/UFL ratios and log2FC values. Because authentic standards were not used to verify retention time and fragment ions, the detected signals are reported as putatively annotated compounds.

#### 2.2.4 Determination of fecal water content

Fecal water content was assessed at two time points: after model establishment and after the final administration. For each measurement, mice were placed individually in clean cages, and newly excreted feces were collected from each animal. The samples were weighed immediately as wet weight, dried at 110 °C until a constant weight was reached, and then reweighed as dry weight. Fecal water content (%) was calculated using the following formula: (wet weight − dry weight)/wet weight × 100%.

#### 2.2.5 Determination of organ indices

Mice were anesthetized with isoflurane in oxygen using an inhalation anesthesia system (4% isoflurane for induction at an oxygen flow rate of 0.5 L/min; 2.0% isoflurane for maintenance). Retro-orbital blood was collected under anesthesia, after which mice were immediately euthanized by cervical dislocation under deep anesthesia. Death was confirmed by cessation of respiration and heartbeat. The spleen and thymus were excised, cleared of attached fat and fascia, blotted dry, and weighed. Organ indices (%) were calculated as organ weight/body weight × 100%.

#### 2.2.6 Determination of serum D-xylose, 5-HT, VIP, MTL, and GAS levels

Blood samples were allowed to clot at room temperature for 3–4 h and were then centrifuged at 3,000 r/min for 10 min at 4 °C. Serum was collected and used to measure D-xylose, 5-HT, VIP, MTL, and GAS according to the protocols supplied with the corresponding ELISA kits.

#### 2.2.7 Determination of hepatic MDA content and SOD activity

For each mouse, two liver portions of approximately 0.1 g were minced and homogenized on ice with 1 mL of the extraction solution from either the MDA or SOD kit. Homogenates were centrifuged at 8,000 × g for 10 min at 4 °C, and the supernatants were kept on ice. Hepatic MDA content and SOD activity were measured spectrophotometrically according to the kit instructions.

#### 2.2.8 Colonic H&E staining and histopathological observation

Following euthanasia, the abdominal cavity was opened, and the colon was excised. Approximately 1 cm of proximal colon tissue was collected, gently washed with precooled normal saline to remove luminal contents, and fixed in 10% neutral buffered formalin for 24 h. The fixed samples were then processed through routine dehydration, clearing, paraffin embedding, sectioning at 4–6 μm, and hematoxylin-eosin staining. Histopathological assessment was performed under a light microscope, focusing on mucosal integrity, crypt morphology, glandular organization, epithelial status, goblet cell distribution, and inflammatory cell infiltration.

#### 2.2.9 Detection of short-chain fatty acids

Colonic contents were homogenized with precooled ultrapure water or phosphate-buffered saline and then centrifuged under low-temperature conditions. The supernatant was combined with an acidification reagent containing the internal standard, incubated, and centrifuged once more. The clarified supernatant was passed through a 0.22 μm membrane and loaded into injection vials. Acetate, propionate, and butyrate were analyzed by GC-FID on a DB-FFAP capillary column. The column oven was programmed as follows: 80 °C for 1 min, increase to 190 °C at 10 °C/min and hold for 0.5 min, then increase to 240 °C at 40 °C/min and hold for 5 min. The injector and detector were both set at 240 °C, nitrogen served as the carrier gas, and SCFA concentrations were calculated using internal-standard calibration curves.

#### 2.2.10 DNA extraction and 16S rRNA high-throughput sequencing

Colonic contents were aseptically collected, snap-frozen in liquid nitrogen, and stored at −80 °C until DNA extraction (Peng et al., 2026). After pretreatment, microbial genomic DNA was isolated with the OMEGA Soil DNA Kit (D5635-02), and DNA integrity and concentration were checked by agarose gel electrophoresis and NanoDrop analysis. The bacterial 16S rRNA V3–V4 region was amplified with primers 338F (5′-ACTCCTACGGGAGGCAGCA-3′) and 806R (5′-GGACTACHVGGGTWTCTAAT-3′). PCR products were examined by gel electrophoresis, purified, quantified, and used to construct sequencing libraries with the Illumina TruSeq Nano DNA LT Library Prep Kit. Paired-end sequencing was carried out on an Illumina NovaSeq 6000 platform by Shanghai Personal Biotechnology Co., Ltd. Five samples from each group were included in the 16S rRNA sequencing analysis. The raw sequencing reads were deposited in the NCBI Sequence Read Archive under BioProject accession number PRJNA1456319.

### 2.3 Bioinformatics analysis

Raw paired-end reads were first demultiplexed, quality-filtered with fastp, trimmed with Cutadapt, merged with FLASH, and screened for chimeras. ASVs were generated using the DADA2 module in QIIME 2 (v2022.2), and taxonomic assignment was performed against SILVA 138.1. Microbial community structure was assessed using alpha diversity, PCoA, NMDS, taxonomic profiles, Venn diagrams, LEfSe, and random forest analysis (Sun et al., 2026a). PICRUSt2 (v2.3.0) was used for functional inference, and KO and pathway annotations were mapped to KEGG. Spearman correlation analysis and RDA were then used to relate genus-level taxa to fecal water content, SCFAs, and host functional indices.

### 2.4 Statistical analysis

Statistical processing was carried out with IBM SPSS Statistics 25.0, and data are reported as mean ± standard deviation. Variables meeting the assumptions of normal distribution and homogeneity of variance were compared by one-way ANOVA with LSD post hoc testing, whereas variables not meeting these assumptions were analyzed using the Kruskal–Wallis *H* test. A two-sided *p* < 0.05 was taken as statistically significant. For LC-MS feature-level comparisons, *q* values refer to false-discovery-rate-adjusted *p* values.

## 3 Results

### 3.1 *A. oryzae* fermentation alters the relative composition of representative putatively annotated compounds in MMF

**Figure 1:**
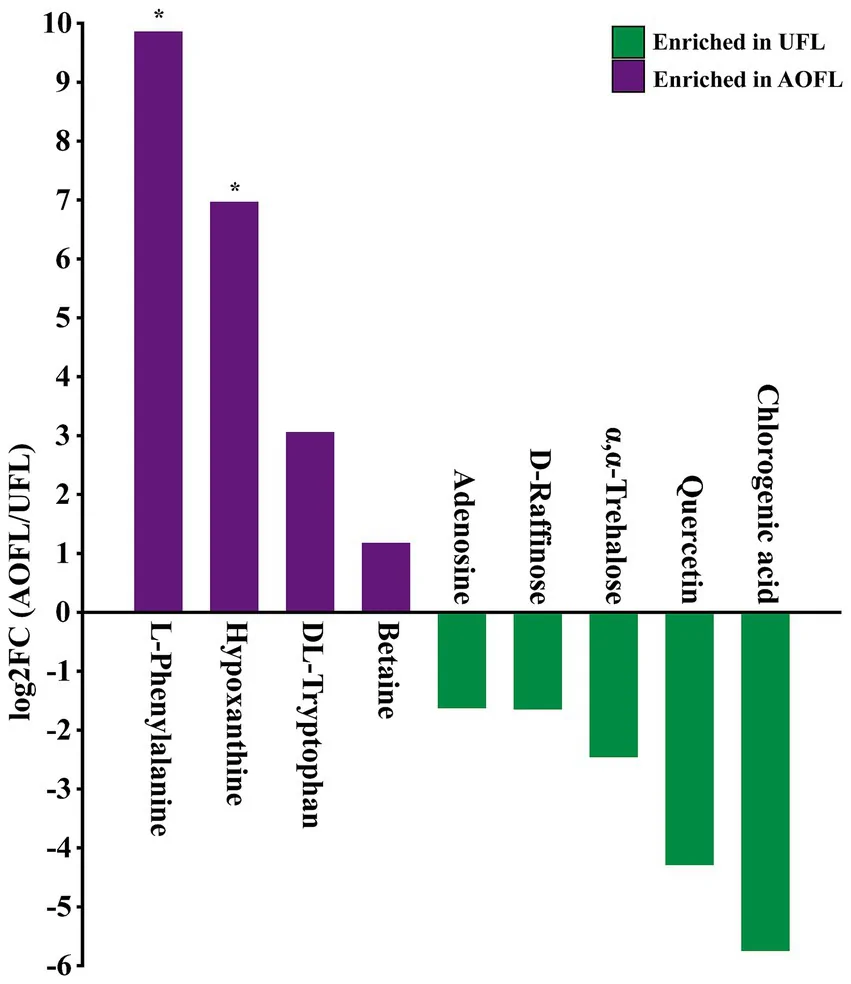
Log2 fold changes (AOFL/UFL) of nine representative putatively annotated compounds in unfermented MMF (UFL) and *Aspergillus oryzae*-fermented MMF (AOFL). Positive values indicate higher relative signal intensities in AOFL, whereas negative values indicate higher relative signal intensities in UFL. *q* values represent multiple-testing-adjusted *p* values obtained from the untargeted LC–MS feature-level analysis. *: *q* < 0.05. Data are presented for three biological replicate samples per group (*n* = 3).

Untargeted LC–MS was used to compare putatively annotated chemical features between UFL and AOFL. Figure 1 summarizes nine representative candidate compounds showing positive or negative log2 fold changes between AOFL and UFL. L-phenylalanine (*q* = 0.007) and hypoxanthine (*q* = 0.034) showed significantly higher relative signal intensities in AOFL than in UFL. DL-tryptophan and betaine also showed positive log2 fold changes, whereas adenosine, D-raffinose, α,α-trehalose, quercetin, and chlorogenic acid showed negative log2 fold changes; however, these differences did not meet the *q* < 0.05 threshold. In terms of chemical classes, the representative features involved amino acid-related, purine/nucleoside-related, sugar-related, and polyphenol-related candidate compounds. This bidirectional pattern suggests that *A. oryzae* fermentation remodeled, rather than uniformly increased, the putatively annotated small-molecule profile of MMF.

### 3.2 Effects of unfermented and *A. oryzae*-fermented MMF on basic phenotypes in model mice

#### 3.2.1 Changes in body weight across groups

**Figure 2:**
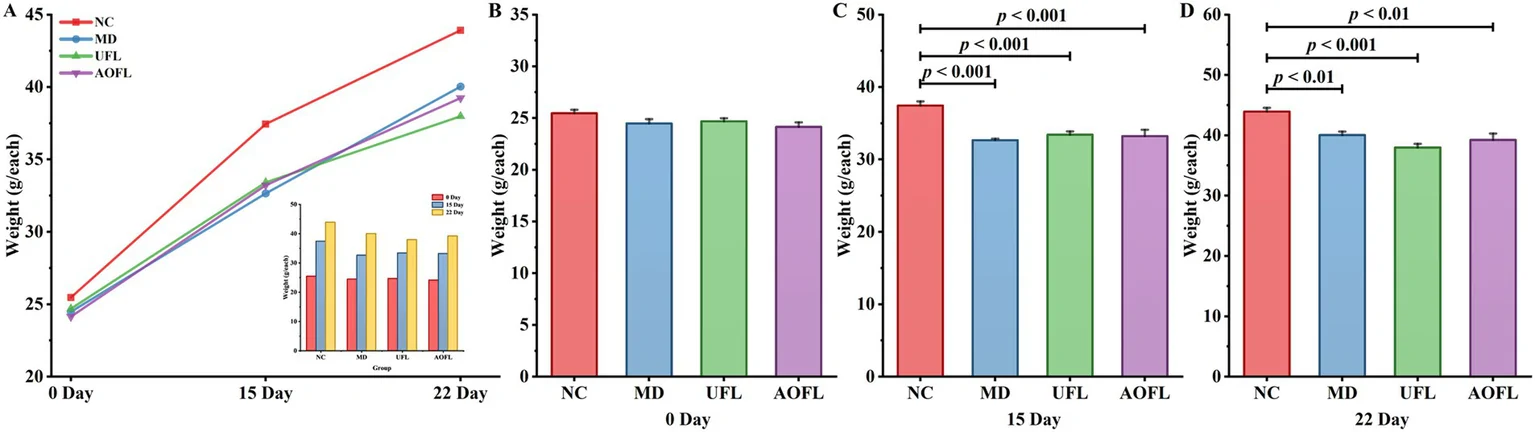
Changes in body weight across groups during the experiment. **(A)** Changes in body weight across groups; **(B)** body weight before the experiment; **(C)** body weight after model establishment; **(D)** body weight after administration.

Figure 2 presents the body-weight changes. Baseline body weight, recorded after random allocation and before model induction, did not differ significantly among the four groups, indicating comparable starting conditions. Body weight increased throughout the experiment in all groups. On day 15, before intervention, the MD, UFL, and AOFL groups had significantly lower body weights than the NC group (*p* < 0.001), indicating a modeling-associated deficit in weight gain. From day 15 to day 22, all groups continued to gain weight after standard chow and unrestricted water access were restored. However, the MD, UFL, and AOFL groups remained significantly lighter than the NC group (*p* < 0.01) on day 22, indicating that the pre-existing growth deficit was not fully compensated during the 7-d intervention period. No significant differences were observed among the MD, UFL, and AOFL groups on day 22.

#### 3.2.2 Changes in spleen and thymus indices across groups

**Figure 3:**
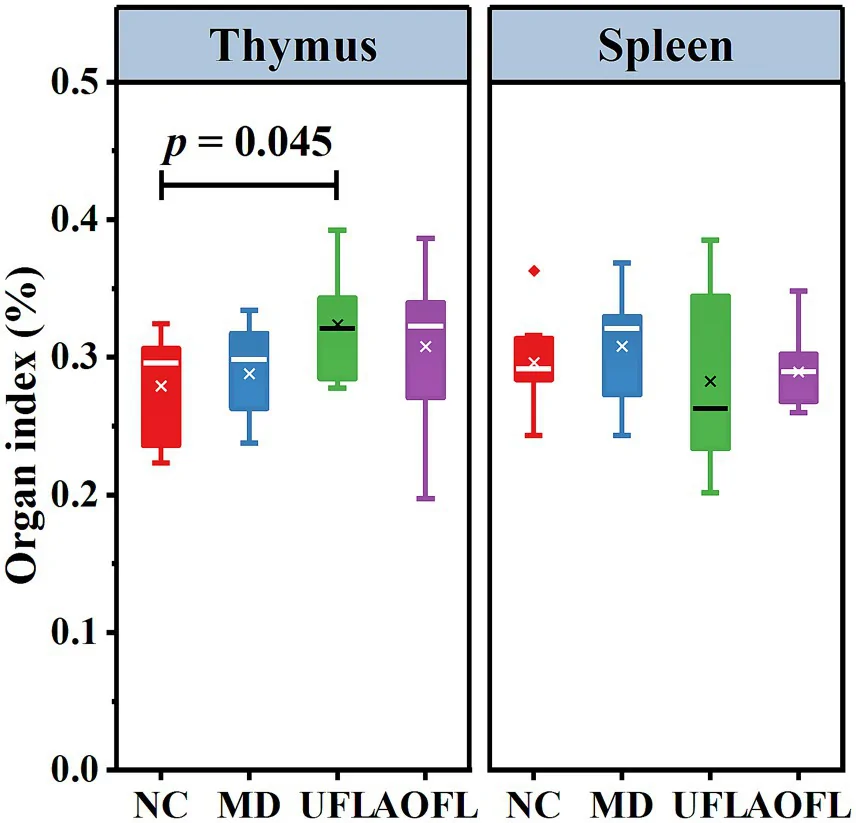
Changes in thymus and spleen indices across groups.

Figure 3 shows thymus and spleen indices. Thymus index tended to increase in the MD and AOFL groups relative to NC, although the differences were not significant. UFL showed a significant increase in thymus index compared with NC (*p* = 0.045). Spleen index changed only slightly in MD mice, and the AOFL value was close to the NC value; no significant difference in spleen index was detected among the groups.

#### 3.2.3 Effects of unfermented and *A. oryzae*-fermented MMF on fecal water content and general condition

**Figure 4:**
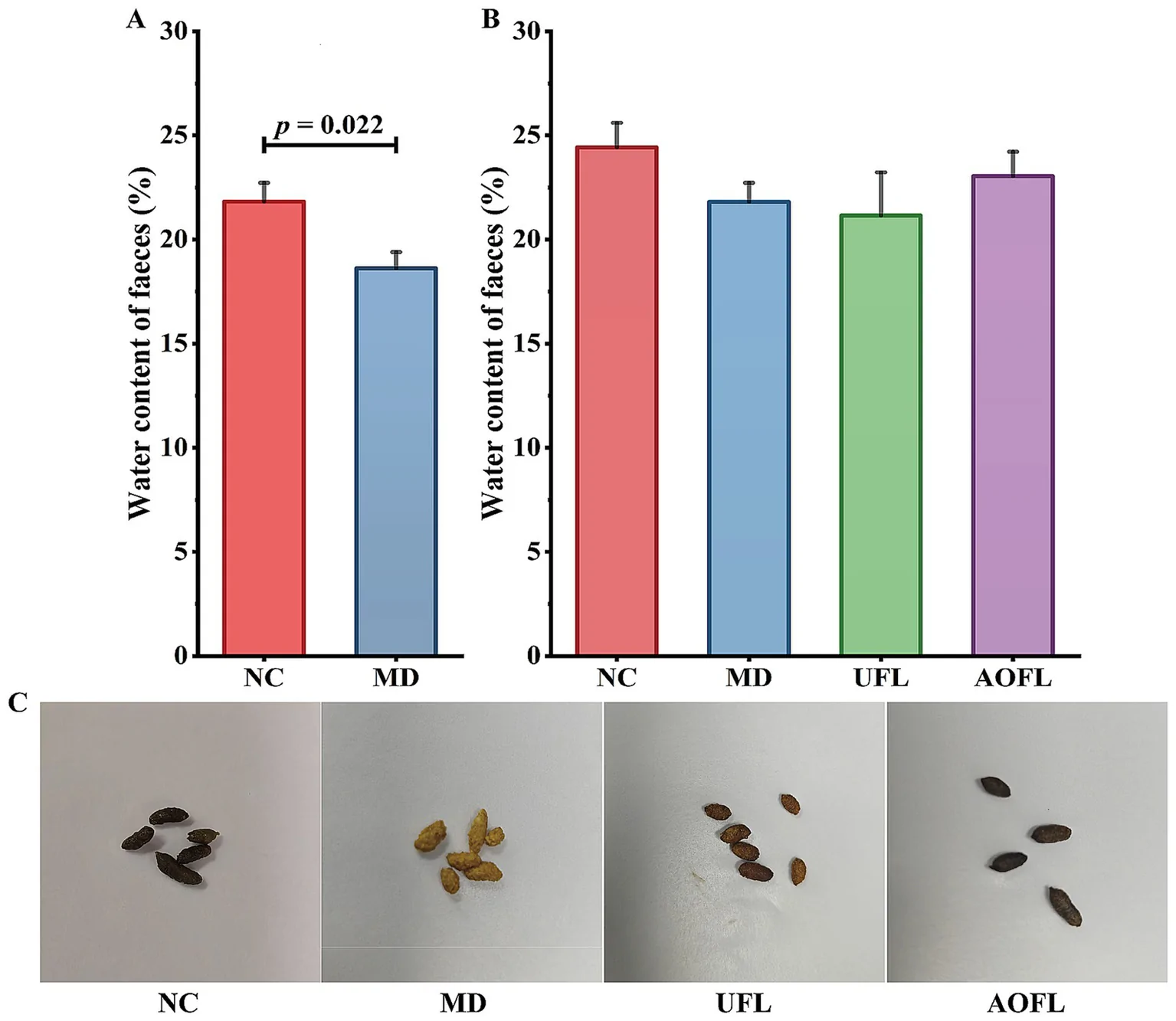
Effects of unfermented and *Aspergillus oryzae*-fermented MMF on fecal water content and fecal appearance. **(A)** Fecal water content in the NC and MD groups after model establishment. **(B)** Fecal water content in the NC, MD, UFL, and AOFL groups after the intervention. **(C)** Representative photographs of fecal pellets collected from the four groups after the intervention.

Figure 4 summarizes fecal water content. After modeling, the MD group had significantly lower fecal water content than the NC group (*p* = 0.022), supporting the establishment of the dry-stool component of the model (Figure 4A). After treatment, values in the MD and UFL groups remained below the NC level, whereas that in the AOFL group was numerically higher than those in the MD and UFL groups and was closer to the NC level; however, no significant differences were detected among the four groups (Figure 4B). Routine observations were consistent with the model phenotype. NC mice remained active and had glossy fur, whereas MD mice showed dull fur and reduced activity. Representative post-intervention photographs showed dark, regularly formed fecal pellets in the NC group and lighter-colored, more irregular fecal pellets in the MD group (Figure 4C). Compared with the MD group, the UFL and AOFL groups showed partial changes in fecal color, form, and apparent texture, with the fecal appearance in the AOFL group more closely resembling that of the NC group.

### 3.3 Effects of unfermented and *A. oryzae*-fermented MMF on intestinal absorptive function and gastrointestinal motility-related factors

**Figure 5:**
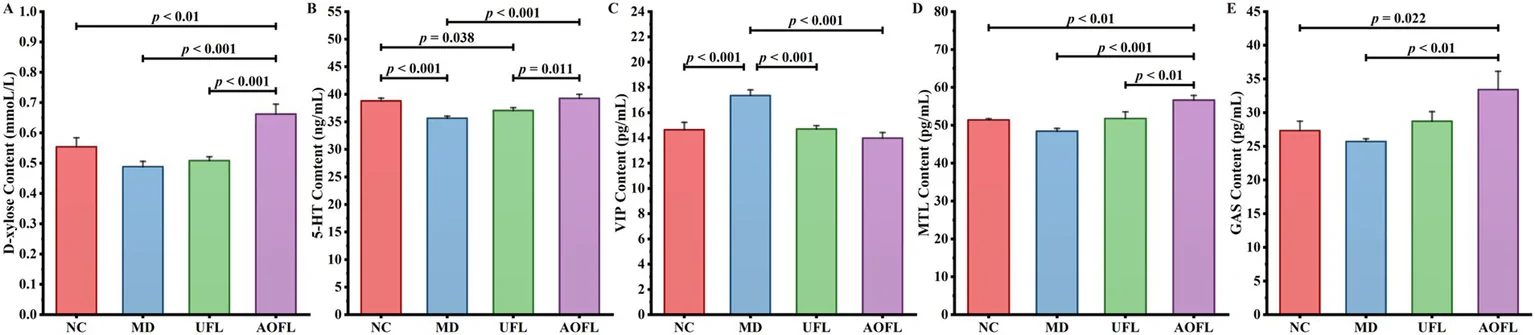
Serum levels of D-xylose and gastrointestinal motility-related factors across groups. **(A)** Serum D-xylose level across groups; **(B)** serum 5-HT level across groups; **(C)** serum VIP level across groups; **(D)** serum MTL level across groups; **(E)** serum GAS level across groups.

Figure 5 presents serum D-xylose, 5-HT, VIP, MTL, and GAS. Compared with NC mice, MD mice showed a downward trend in D-xylose, a marked decrease in 5-HT (*p* < 0.001), and an increase in VIP (*p* < 0.001). AOFL significantly increased D-xylose compared with NC, MD, and UFL (*p* < 0.01). AOFL also increased 5-HT relative to MD (*p* < 0.001) and UFL (*p* = 0.011), and decreased VIP relative to MD (*p* < 0.01). In addition, MTL was higher in AOFL than in NC, MD, and UFL (*p* < 0.01), while GAS was higher than in NC (*p* = 0.022) and MD (*p* < 0.01).

### 3.4 Effects of unfermented and *A. oryzae*-fermented MMF on oxidative stress status and colonic mucosal structure

#### 3.4.1 Effects of unfermented and *A. oryzae*-fermented MMF on hepatic MDA content and SOD activity

**Figure 6:**
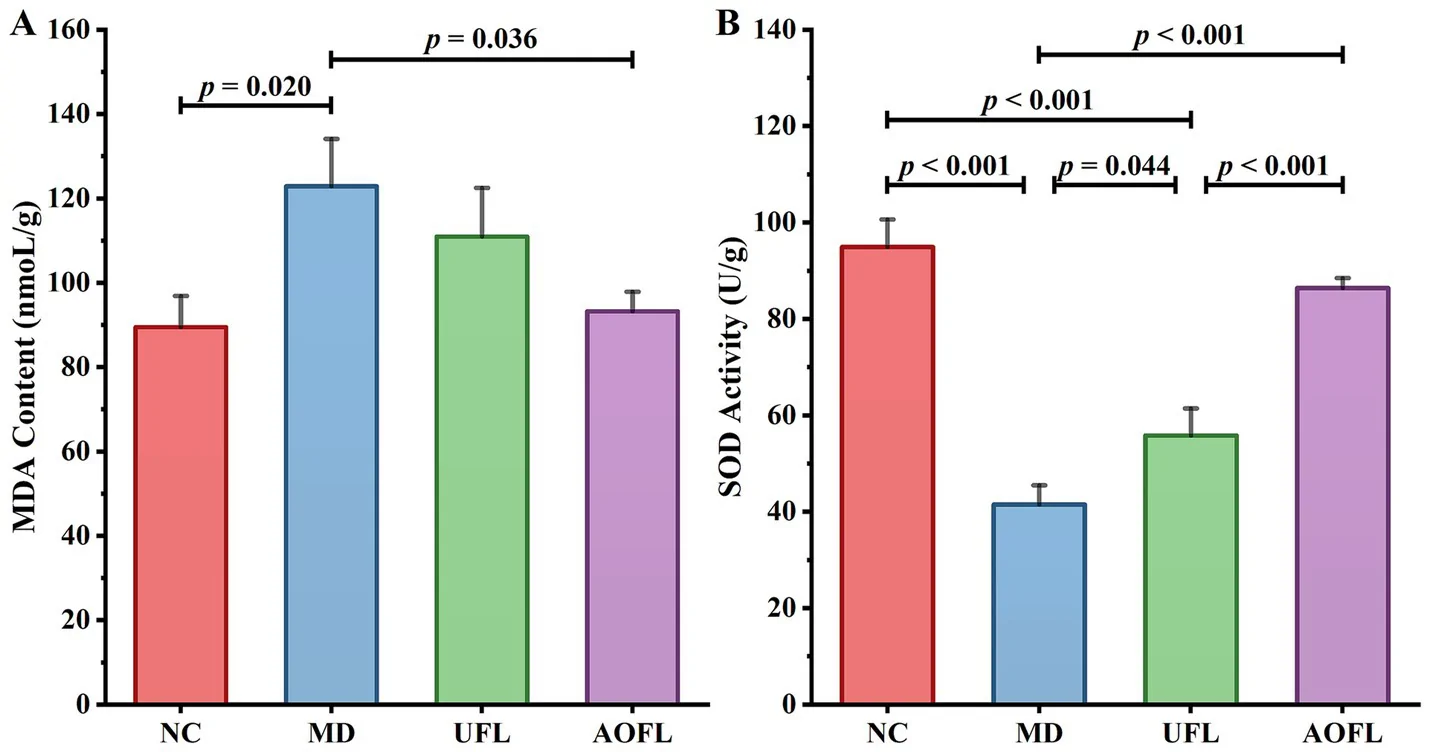
Changes in hepatic MDA content and SOD activity across groups. **(A)** Hepatic MDA content across groups; **(B)** hepatic SOD activity across groups.

Figure 6 shows hepatic MDA content and SOD activity. Compared with NC mice, MD mice had higher MDA (*p* = 0.020) and lower SOD activity (*p* < 0.001), indicating increased oxidative stress after modeling. UFL tended to lower MDA relative to MD, but the change was not significant. AOFL significantly reduced MDA compared with MD (*p* = 0.036). SOD activity increased in both the UFL (*p* = 0.044) and AOFL (*p* < 0.001) groups relative to MD, and AOFL produced higher SOD activity than UFL (*p* < 0.001).

#### 3.4.2 Effects of unfermented and *A. oryzae*-fermented MMF on colonic mucosal structure, crypt length, and goblet cell number

**Figure 7:**
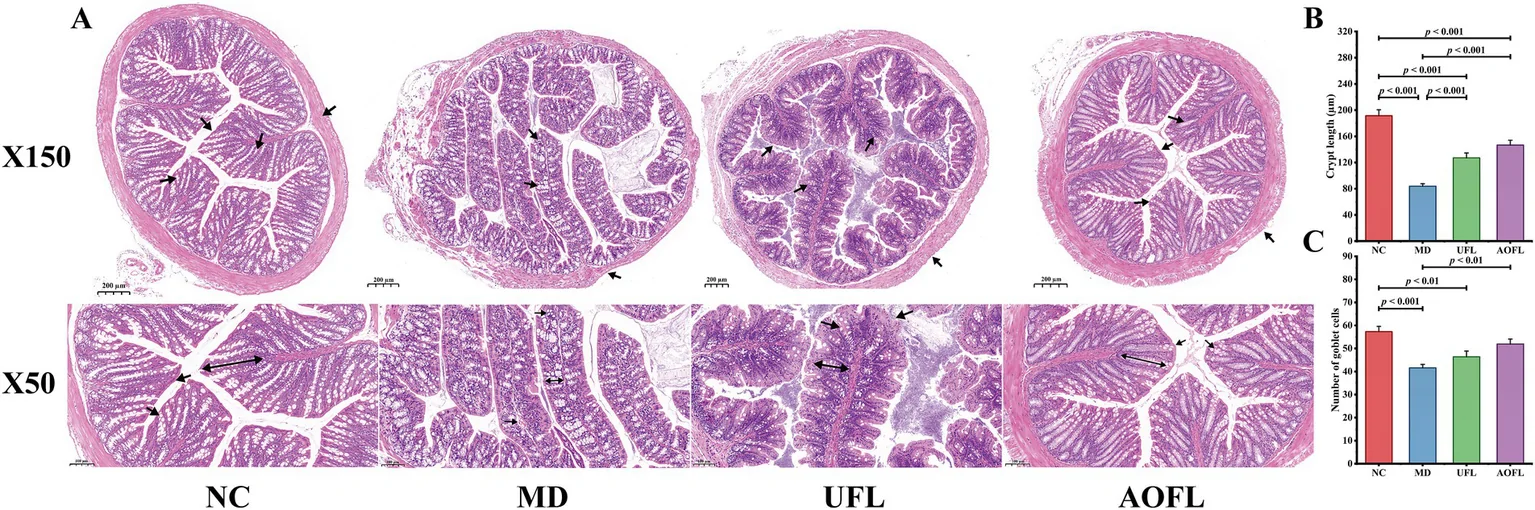
Changes in colonic morphology, crypt length, and goblet cell number across groups. **(A)** H&E-stained sections; **(B)** crypt length; **(C)** goblet cell number.

Figure 7 shows representative H&E sections and quantitative histological indices. NC mice had an intact mucosa, regular crypt arrangement, and abundant goblet cells. MD mice showed mucosal injury, shortened crypts, disorganized glands, and fewer goblet cells. Both UFL and AOFL improved the histological appearance relative to MD, with clearer recovery in the AOFL group (Figure 7A). Crypt length was shorter in MD mice than in NC mice (*p* < 0.001), but increased after UFL and AOFL treatment (*p* < 0.001 versus MD). AOFL further increased crypt length compared with UFL (*p* < 0.001), although neither intervention restored it to the NC level (*p* < 0.001). Goblet cell number decreased in MD mice (*p* < 0.001), and AOFL increased this value relative to MD (*p* < 0.01), but it remained below NC (*p* < 0.01).

### 3.5 Effects of unfermented and *A. oryzae*-fermented MMF on the concentrations of SCFAs in colonic contents

**Figure 8:**
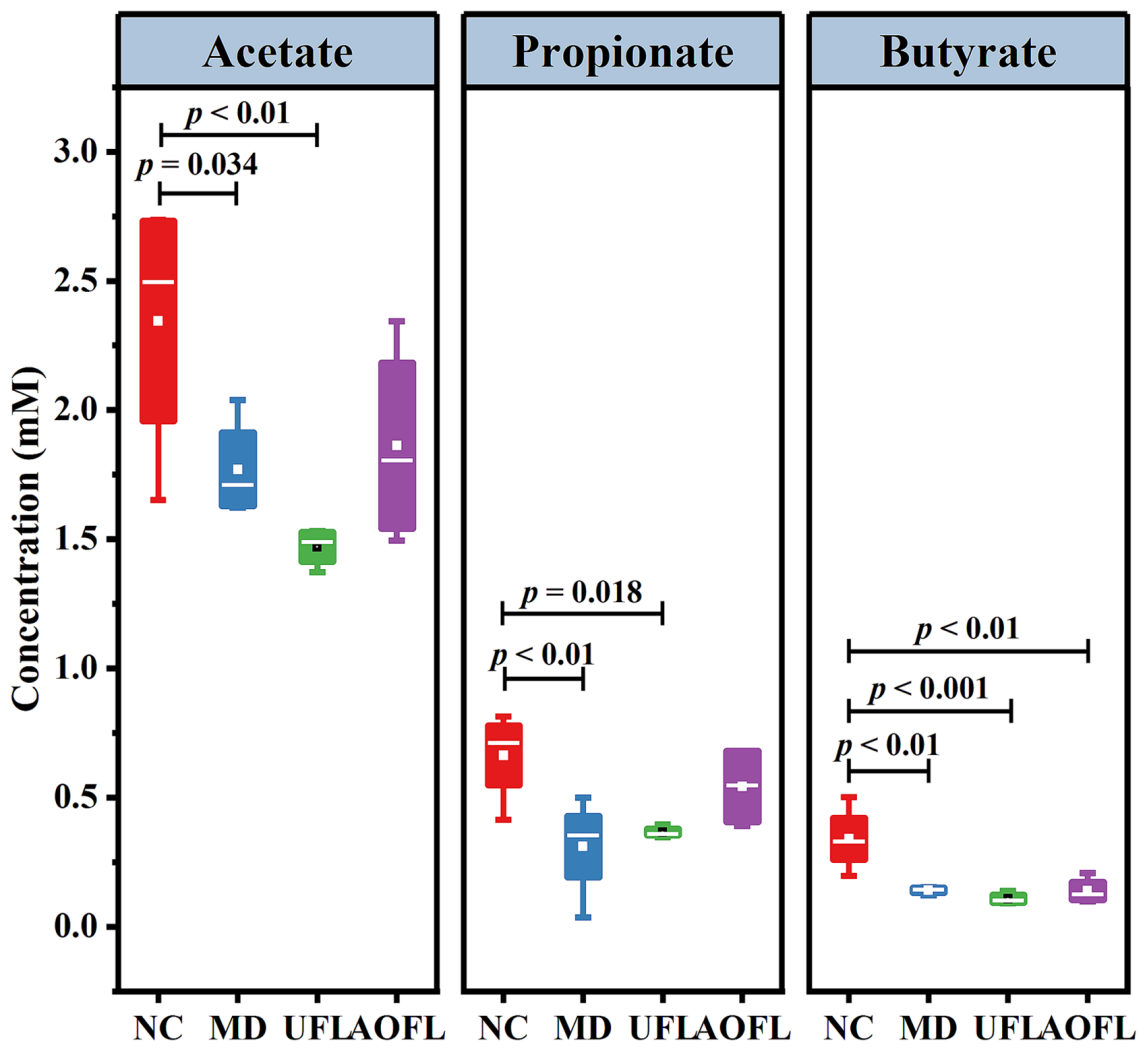
Levels of acetate, propionate, and butyrate in colonic contents across groups.

Figure 8 presents acetate, propionate, and butyrate levels in colonic contents. Acetate was lower in the MD group than in the NC group (*p* = 0.034) and was further decreased in the UFL group compared with NC group (*p* < 0.01). The acetate level in the AOFL group was numerically higher than that in the MD group and was intermediate between the MD and NC groups, but the difference relative to MD was not statistically significant. Propionate was also lower in MD than in NC (*p* < 0.01), and the UFL group remained below NC (*p* = 0.018). Propionate was numerically higher in the AOFL group than in the MD and UFL groups, but the differences were not statistically significant. Butyrate remained lower than NC in the MD (*p* < 0.01), UFL (*p* < 0.001), and AOFL (*p* < 0.01) groups. Thus, AOFL did not significantly restore the measured SCFAs; acetate and propionate showed only compound-specific numerical differences, whereas butyrate did not show a corresponding recovery.

### 3.6 Effects of unfermented and *A. oryzae*-fermented MMF on microbial structure and predicted functions in colonic contents

#### 3.6.1 Effects of unfermented and *A. oryzae*-fermented MMF on microbial diversity in colonic contents

**Figure 9:**
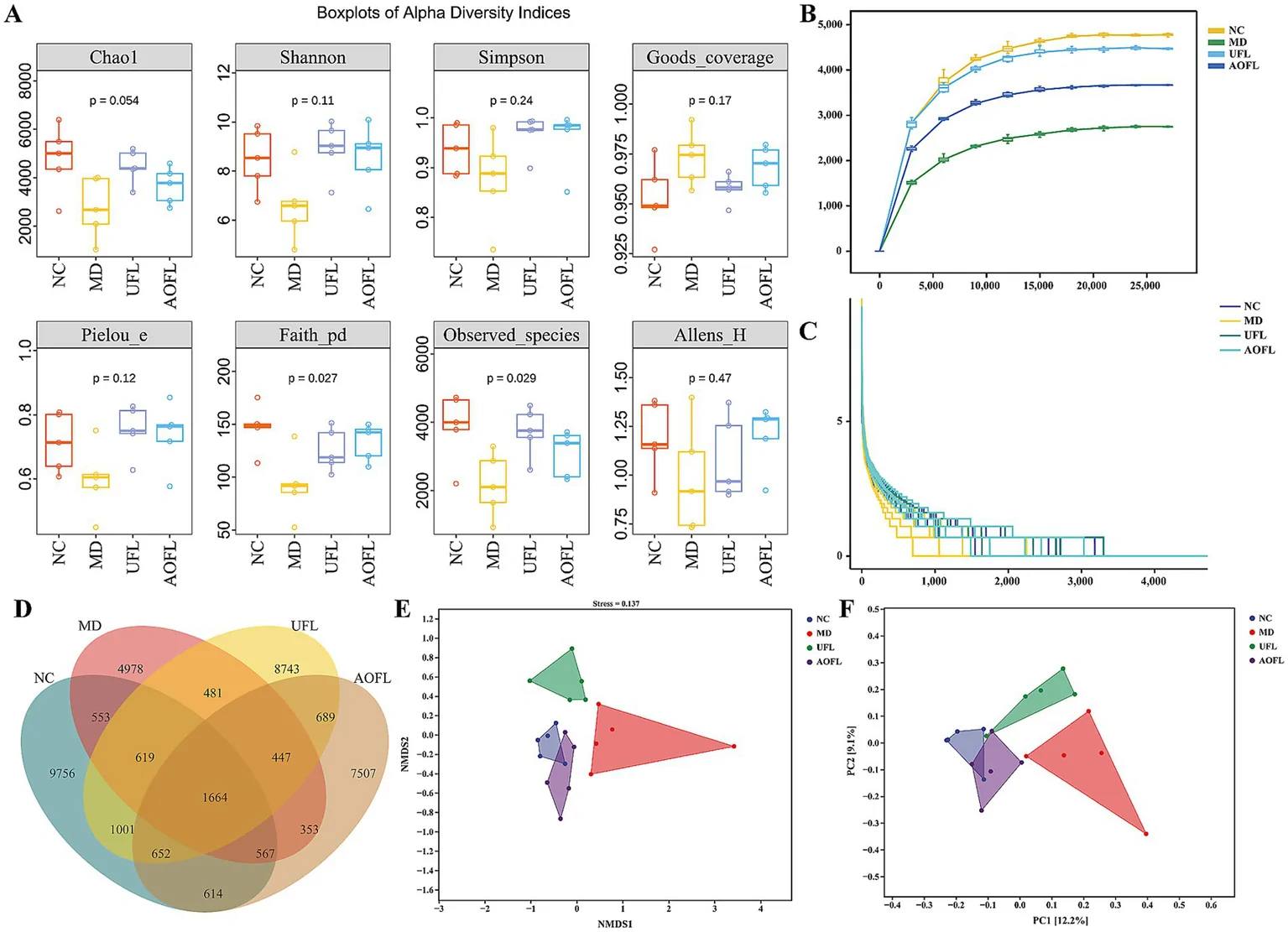
Analysis of α-diversity and β-diversity of microbial communities in colonic contents across groups. **(A)** Alpha diversity indices; **(B)** rarefaction curves; **(C)** rank abundance curves; **(D)** Venn diagram; **(E)** NMDS analysis; **(F)** PCoA analysis.

Figure 9 shows alpha- and beta-diversity results. Compared with NC, the MD group showed downward trends in Chao1, Faith_pd, and Observed_species, with significant decreases in Faith_pd (*p* = 0.027) and Observed_species (*p* = 0.029), suggesting reduced richness after modeling. These indices increased in the UFL and AOFL groups relative to MD (Figure 9A). Rarefaction and rank-abundance curves supported sufficient sequencing depth and species-distribution coverage (Figures 9B,C). The Venn diagram showed 1664 ASVs shared across all groups, together with group-specific ASVs (Figure 9D). Beta-diversity plots separated MD from NC, whereas UFL and AOFL shifted toward NC; this shift was more evident for AOFL (Figures 9E,F).

#### 3.6.2 Effects of unfermented and *A. oryzae*-fermented MMF on microbial composition and differential taxa in colonic contents

**Figure 10:**
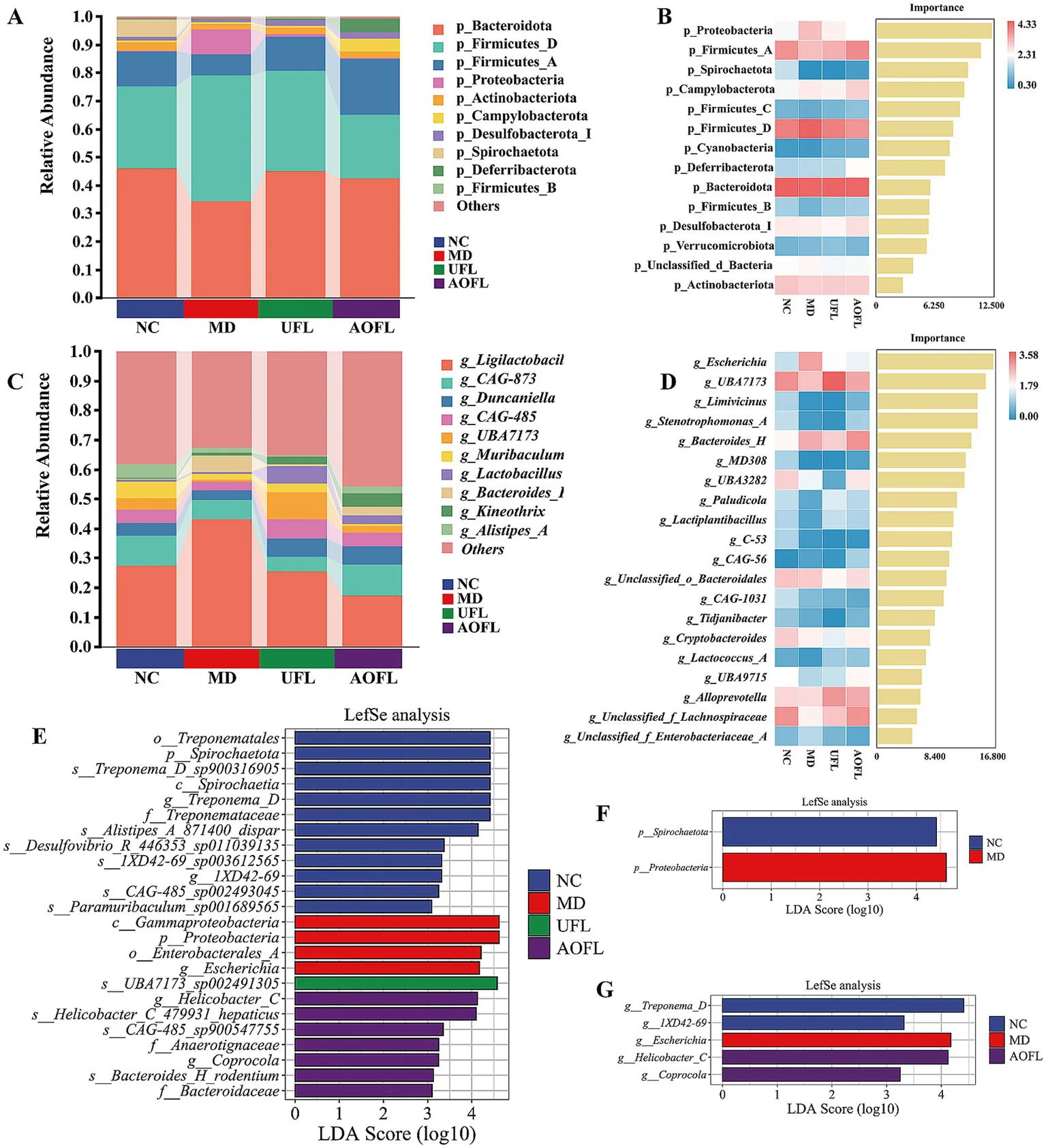
Analysis of microbial composition and differential taxa in colonic contents across groups. **(A)** Taxonomic composition at the phylum level; **(B)** random forest analysis at the phylum level; **(C)** taxonomic composition at the genus level; **(D)** random forest analysis at the genus level; **(E)** LEfSe analysis across all taxonomic levels; **(F)** LEfSe analysis at the phylum level; **(G)** LEfSe analysis at the genus level.

Figure 10 summarizes taxonomic composition, random forest analysis, and LEfSe results. At the phylum level, Bacteroidota, Firmicutes_D, Firmicutes_A, Proteobacteria, Actinobacteriota, Campylobacterota, and Spirochaetota were the main groups (Figure 10A). Relative to NC, MD mice had lower Bacteroidota and higher Firmicutes_D. UFL increased Bacteroidota, and AOFL also changed the phylum-level profile. Random forest analysis at the phylum level selected Proteobacteria, Firmicutes_A, Spirochaetota, Campylobacterota, Firmicutes_C, Firmicutes_D, Cyanobacteria, and Deferribacterota as important discriminating variables (Figure 10B). At the genus level, the dominant taxa included *Ligilactobacil*, *CAG-873*, *Duncaniella*, *CAG-485*, *UBA7173*, *Muribaculum*, *Lactobacillus*, *Bacteroides_I*, *Kineothrix*, and *Alistipes_A* (Figure 10C). MD mice showed higher relative abundance of *Ligilactobacil* and changes in several other genera. The genus-level random forest model identified *Escherichia*, *UBA7173*, *Limivicinus*, *Stenotrophomonas_A*, *Bacteroides_H*, *MD308*, *UBA3282*, *Paludicola*, and *Lactiplantibacillus* as key taxa for group discrimination (Figure 10D).

LEfSe analysis identified group-characteristic taxa (LDA ≥ 2, Figure 10E). NC samples were characterized by Treponematales, Spirochaetota, *Treponema_D*, Treponemataceae, *Alistipes_A*, *Desulfovibrio_R*, *IXD42-69*, and *CAG-485*. MD samples were characterized by Campylobacterota, Proteobacteria, Enterobacterales, and *Escherichia*. UFL was mainly associated with *UBA7173*. AOFL showed enrichment of *Helicobacter_C*, *Helicobacter_C*_479931_hepaticus, *CAG-485*, Anaerotignaceae, *Coprocola*, *Bacteroides_H_rodentium*, and Bacteroidaceae. Across taxonomic levels, Spirochaetota was the main NC-associated phylum, whereas Proteobacteria was the main MD-associated phylum (Figure 10F). At the genus level, *Treponema_D* and *IXD42-69* characterized NC, *Escherichia* characterized MD, and *Helicobacter_C* and *Coprocola* characterized AOFL (Figure 10G). These findings indicate that modeling altered gut microbial composition, with Proteobacteria and *Escherichia* enrichment, and that MMF intervention partly reshaped the community, especially after *A. oryzae* fermentation.

#### 3.6.3 Effects of unfermented and *A. oryzae*-fermented MMF on predicted microbial functions in colonic contents

**Figure 11:**
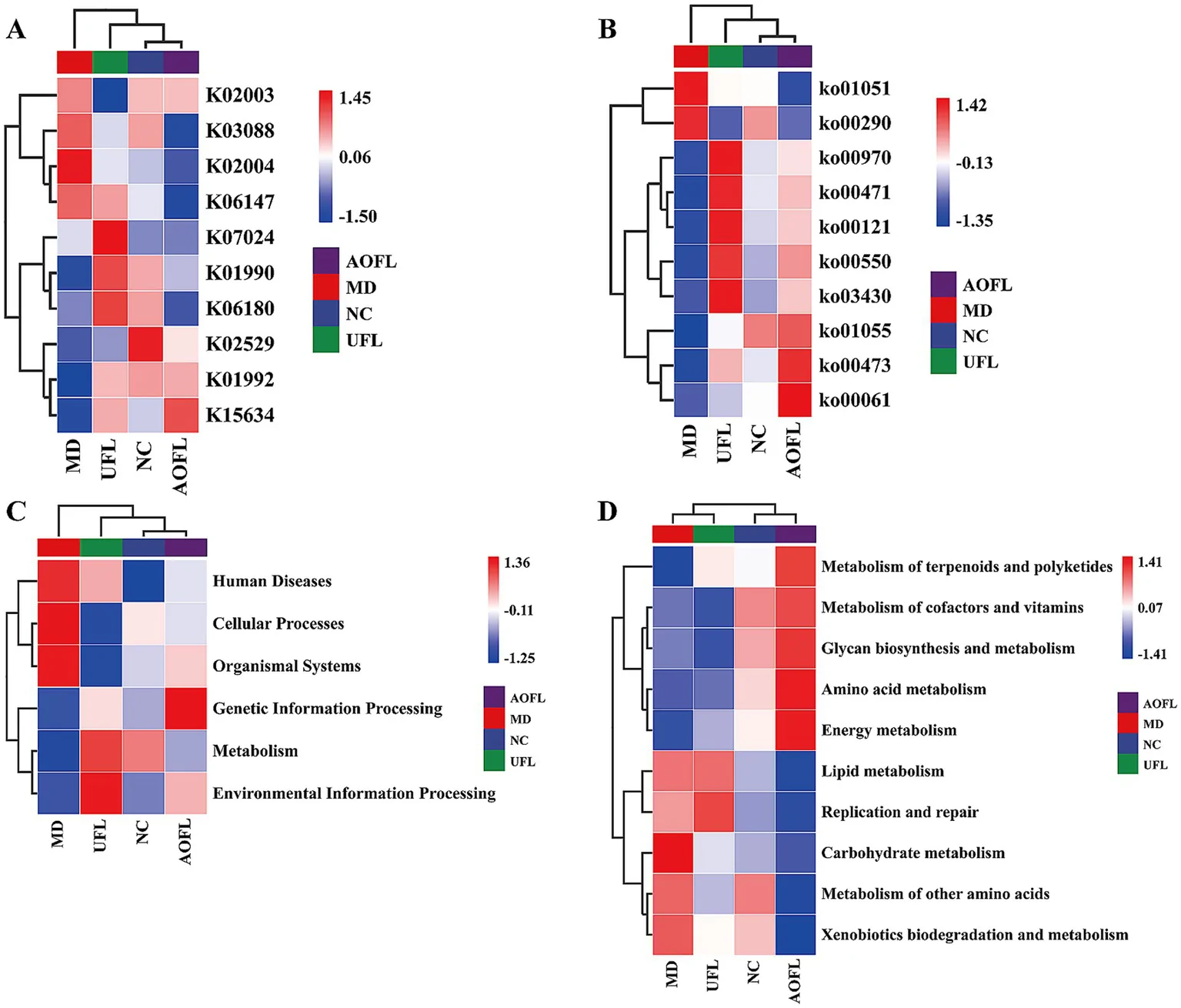
PICRUSt2-based functional prediction analysis of microbial communities in colonic contents across groups. **(A)** KEGG KO analysis; **(B)** KEGG pathway analysis; **(C)** KEGG level 1 analysis; **(D)** KEGG level 2 analysis.

Figure 11 presents predicted microbial functions. PICRUSt2 indicated distinct distributions among groups at the KO and KEGG pathway levels (Figures 11A,B). K02004, ko01051, and ko00290 were relatively higher in MD and lower in AOFL. K07024, ko00970, ko00471, ko00121, ko00550, and ko03430 were higher in UFL, whereas K15634, ko00473, and ko00061 were higher in AOFL. KEGG level 1 and level 2 summaries also differed across groups, particularly in Metabolism, Genetic Information Processing, and Environmental Information Processing. The AOFL group showed higher relative abundance in several metabolism-related categories, including metabolism of cofactors and vitamins, glycan biosynthesis and metabolism, amino acid metabolism, and energy metabolism (Figures 11C,D). These results suggest that AOFL partially modifies the predicted functional composition of the microbiota in model mice.

#### 3.6.4 Correlation analysis among colonic microbiota, constipation-related phenotypes, SCFAs, and host functional indices

**Figure 12:**
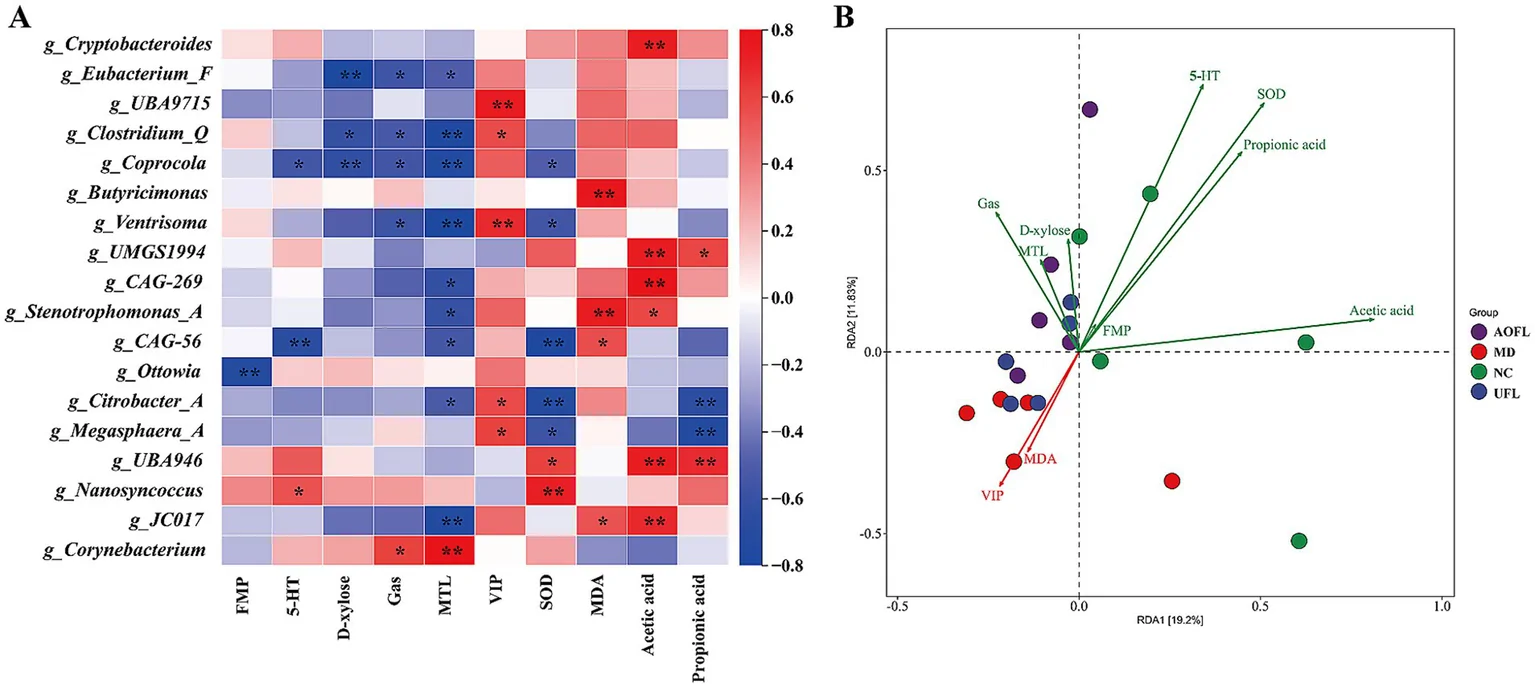
Correlation analysis of genus-level microbiota with constipation-related phenotypes, SCFAs, and host functional indices. **(A)** Correlation heatmap; **(B)** RDA analysis.

Spearman correlation analysis revealed coordinated associations between genus-level microbial taxa and constipation-related phenotypes, host functional indices, and selected SCFAs (Figure 12A). A recurring pattern was observed for *Coprocola*, *Ventrisoma*, *Citrobacter_A*, *Megasphaera_A*, *CAG-56*, and *Clostridium_Q*: these genera were negatively correlated with one or more indices related to intestinal absorption, motility, or antioxidant status, including D-xylose, 5-HT, MTL, and SOD, whereas several were positively correlated with VIP or MDA. In contrast, *UBA946* and *Nanosynococcus* were positively correlated with SOD and 5-HT. *UBA946*, *UMGS1994*, *Cryptobacteroides*, *CAG-269*, *Stenotrophomonas_A*, and *JC017* were positively correlated with acetate and/or propionate, whereas *Citrobacter_A* and *Megasphaera_A* were negatively correlated with propionate. *Ottowia* showed a distinct negative correlation with fecal water content, whereas *Corynebacterium* was positively correlated with GAS and MTL. These findings indicate that genus-level microbial variation was associated with multiple functional domains rather than with a single constipation-related endpoint.

RDA1 and RDA2 explained 19.2% and 11.83% of the genus-level community variation, respectively (Figure 12B). MD samples were mainly distributed in the negative RDA1 and RDA2 directions, broadly corresponding to the VIP and MDA vectors. NC samples extended toward the positive RDA1 and RDA2 regions, in the directions of acetate, 5-HT, SOD, and propionate. AOFL samples extended away from the MD distribution and toward the positive RDA2 region, broadly aligning with D-xylose, GAS, MTL, 5-HT, and SOD, whereas UFL samples were mainly located near the origin or on the negative RDA1 side. However, overlap among groups remained, indicating partial rather than complete separation. Taken together, the correlation and ordination analyses suggest coordinated variation among microbial community structure, intestinal absorptive and motility-related indices, oxidative-stress status, and acetate and propionate.

## 4 Discussion

**Figure 13:**
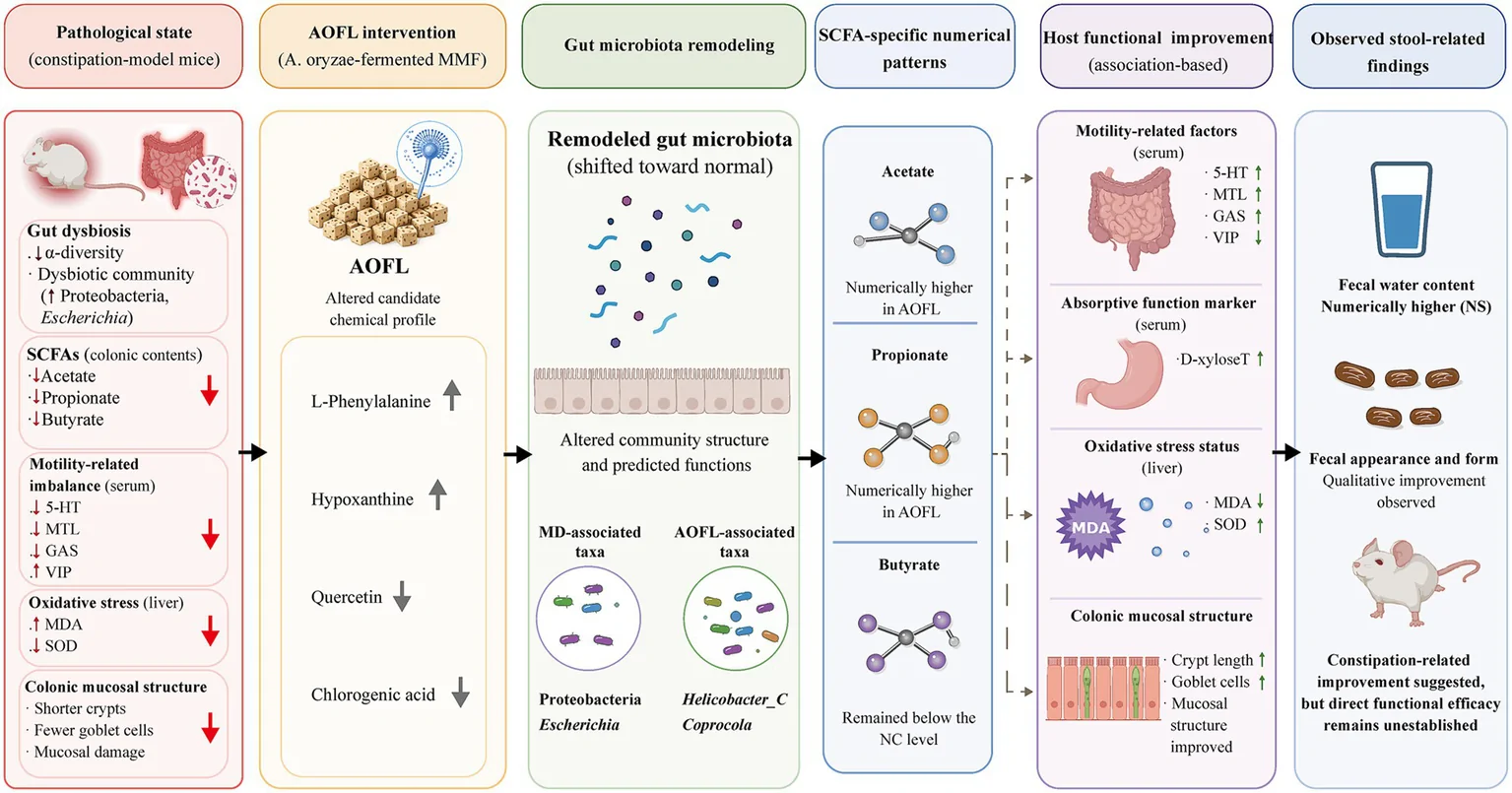
Proposed association network linking *A. oryzae*-fermented MMF, gut microbiota, SCFAs, and selected host-response indices in a mouse model of spleen-deficiency constipation.

This study directly compared UFL and AOFL to evaluate the effects of defined *A. oryzae* fermentation on the putatively annotated chemical composition of MMF, gut microbial composition, and selected host-response indices in mice with spleen-deficiency constipation. Fermentation remodeled the relative profile of representative putatively annotated LC–MS features. Compared with UFL, AOFL showed more pronounced changes in selected intestinal absorptive-function and motility-related serum indices, hepatic oxidative-stress indices, colonic mucosal structure, and gut microbial composition. However, none of the measured SCFAs differed significantly between the AOFL and MD groups. Overall, the findings are consistent with an integrated chemical–microbial–host framework for fermented medicinal materials (Figure 13), but they do not establish microbiota- or SCFA-mediated causality (Ríos-Covián et al., 2016; Liu et al., 2025; Ma et al., 2025).

### 4.1 *A. oryzae* fermentation remodels the relative profile of representative putatively annotated compounds in MMF

The effects of MMF depend not only on its original medicinal materials but also on substrate degradation and transformation during fermentation. *A. oryzae* can secrete extracellular hydrolases, including amylases, proteases, and glycosidases, which promote the hydrolysis of starch, proteins, and glycosides. Together with fungal substrate utilization and primary metabolism, these enzymatic processes can alter amino acids, sugars, organic acids, nucleosides, and other small molecules during fermentation (Daba et al., 2021; Sun et al., 2024).

In the present study, untargeted LC–MS analysis showed bidirectional differences among nine representative putatively annotated features. L-phenylalanine and hypoxanthine had significantly higher relative signal intensities in AOFL than in UFL, whereas DL-tryptophan and betaine showed nonsignificant positive log2 fold changes. Adenosine, D-raffinose, α,α-trehalose, quercetin, and chlorogenic acid showed nonsignificant negative log2 fold changes. The higher signals of selected amino acid- and purine-related candidates may be associated with protein hydrolysis and purine/nucleoside turnover, whereas the lower numerical signals of selected sugars and polyphenol-related candidates may reflect fungal consumption or enzymatic transformation (Li et al., 2023). Thus, *A. oryzae* fermentation appeared to remodel, rather than uniformly increase, the putatively annotated small-molecule profile of MMF.

Such chemical remodeling may alter the carbon sources, nitrogen sources, and plant-derived substrates available to the gut microbiota after AOFL administration (Ríos-Covián et al., 2016; Portincasa et al., 2022). However, enzyme activities and compound-transformation pathways were not directly measured, and the LC–MS annotations were not confirmed using authentic standards. Therefore, the chemical data provide a plausible upstream context rather than evidence that individual compounds mediated specific microbial or host responses (Sumner et al., 2007).

### 4.2 AOFL is associated with more pronounced changes in intestinal absorptive-function and motility-related serum indices than UFL

Spleen-deficiency constipation is not limited to abnormal defecation; it is also often accompanied by impaired digestive and intestinal absorptive function and dysregulated gastrointestinal motility (Yu et al., 2022). The D-xylose absorption test is commonly used to evaluate small intestinal absorptive function. In animal models related to spleen deficiency, decreased D-xylose levels usually indicate weakened small intestinal absorption (Zhu et al., 2018; You et al., 2020; Yu et al., 2022). In this study, the MD group showed a nonsignificant downward trend in D-xylose compared with the NC group, accompanied by suppressed body-weight gain, reduced fecal water content, and worsened general condition. Together, these findings suggest possible impairment of intestinal absorptive function but do not independently confirm it. Compared with the UFL group, the AOFL group shows a significantly higher D-xylose level, indicating that *A. oryzae*-fermented MMF has a more evident effect on intestinal absorptive function-related indices in model mice. Although the UFL and AOFL groups continued to gain weight during the intervention period, their body weights did not recover to the NC level or differ significantly from that of the MD group on day 22. This may reflect persistence of the difference established during modeling, together with spontaneous recovery in the MD group after standard chow and unrestricted water access were restored. Because food intake, energy intake, and body composition were not quantitatively assessed, the factors underlying the incomplete recovery of body weight remain uncertain.

Gastrointestinal motility is jointly regulated by the enteric nervous system, gastrointestinal hormones, and the local intestinal microenvironment. 5-HT participates in intestinal peristalsis, secretion, and sensory transmission (Sikander et al., 2009; Gershon, 2013; Israelyan et al., 2019). MTL and GAS are generally associated with enhanced gastrointestinal propulsion and digestive function (Wu et al., 2024), whereas VIP is considered an inhibitory non-adrenergic non-cholinergic neurotransmitter involved in gastrointestinal smooth muscle relaxation and motility regulation (Van Geldre and Lefebvre, 2004; Cheng et al., 2023). Because this study measures these indices in serum, they are more appropriately interpreted as systemic reflections of gastrointestinal functional status rather than direct evidence of local enteric nerve or enteroendocrine cell expression. The results show that the MD group has decreased 5-HT and increased VIP, suggesting weakened pro-motility signaling and enhanced inhibitory regulation in model mice. After intervention, the AOFL group shows significantly higher 5-HT and MTL levels than the UFL group, lower VIP than the MD group, and higher GAS than the MD group. These results indicate that *A. oryzae*-fermented MMF has more evident effects on serum markers related to gastrointestinal motility, particularly 5-HT and MTL, than unfermented MMF. Nevertheless, these serum markers cannot substitute for direct measurements of gastrointestinal transit or defecation and therefore do not establish a pro-motility effect of AOFL.

Because UFL and AOFL shared the same basic formulation, the more pronounced changes in D-xylose, 5-HT, and MTL observed with AOFL are consistent with a fermentation-associated difference in host response. However, these serum indices do not directly demonstrate improved gastrointestinal transit, and the present data do not identify the chemical or microbial factors responsible for these differences.

### 4.3 AOFL is associated with more pronounced changes in hepatic oxidative-stress indices and colonic mucosal structure than UFL

Oxidative stress is involved in pathological changes associated with constipation, including gastrointestinal motility dysfunction and barrier injury. Increased MDA usually indicates aggravated lipid peroxidation, whereas decreased SOD activity reflects weakened antioxidant defense. These two indices are commonly used to evaluate tissue oxidative damage and antioxidant status (Mao et al., 2019; Cordiano et al., 2023). Constipation-related studies show that enhanced oxidative stress can affect intestinal smooth muscle, enteric nerves, and mucosal epithelial function, thereby aggravating gastrointestinal motility dysfunction and barrier injury (Yi et al., 2023; Chen et al., 2024). In this study, the MD group shows increased hepatic MDA content and decreased SOD activity, suggesting elevated oxidative stress in model mice. After intervention, MDA content in the UFL group only shows a decreasing trend compared with the MD group, whereas MDA content in the AOFL group is significantly lower than that in the MD group. SOD activity is higher in both the UFL and AOFL groups than in the MD group, and the AOFL group shows significantly higher SOD activity than the UFL group. These results indicate that both MMF preparations attenuate oxidative stress-related changes in model mice to some extent, but *A. oryzae*-fermented MMF has more evident effects on hepatic MDA and SOD indices.

The colonic mucosa and mucus barrier are important structural bases for maintaining intestinal homeostasis. Excessive reactive oxygen species can damage epithelial structure, tight junctions, and the mucus barrier. Mucins such as MUC2 secreted by goblet cells help maintain luminal lubrication and barrier integrity in the intestine (Kim and Ho, 2010; Wang et al., 2020; Yang and Yu, 2021; Rezvani, 2024). In this study, H&E staining shows shortened crypts, disordered glandular arrangement, and reduced goblet cell number in the colonic tissue of the MD group, suggesting colonic mucosal structural injury in model mice (Kim et al., 2020). After intervention, colonic structure is improved in both the UFL and AOFL groups compared with the MD group. In particular, crypt length in the AOFL group is significantly higher than that in the UFL group, and goblet cell number is also significantly higher than that in the MD group, indicating that *A. oryzae*-fermented MMF has a more evident effect on colonic crypt structure than unfermented MMF. These findings characterize systemic oxidative-stress and colonic mucosal responses to AOFL but do not independently demonstrate relief of constipation. These oxidative-stress and mucosal responses occurred alongside microbial restructuring and nonsignificant numerical differences in acetate and propionate; however, crypt length was not included in the correlation analysis, and a microbiota- or SCFA-mediated link remains unproven.

### 4.4 AOFL is associated with distinct gut microbial restructuring and coordinated host-response patterns

Gut microbiota may provide an interface between fermentation-associated chemical changes in MMF and host responses. Substrates that escape small-intestinal digestion can be utilized by colonic microorganisms and converted into metabolites, including SCFAs, with potential effects on intestinal metabolism and barrier homeostasis (den Besten et al., 2013; Ríos-Covián et al., 2016). In this study, the MD group showed reduced microbial richness and a community structure distinct from that of the NC group, accompanied by enrichment of Proteobacteria and *Escherichia*. Expansion of Proteobacteria and facultative anaerobes such as *Escherichia* is commonly associated with disturbed intestinal microecology (Shin et al., 2015; Rizzatti et al., 2017; Zeng et al., 2017; Iancu et al., 2023). Both UFL and AOFL partially shifted the microbial community away from the MD pattern, with AOFL appearing closer to NC in the β-diversity analyses.

AOFL also showed a taxonomic profile distinct from that of UFL, characterized by Bacteroidaceae, *Bacteroides_H_rodentium*, *Coprocola*, and *Helicobacter_C*. Fermentation-associated differences in sugar-, amino acid-, and purine/nucleoside-related candidate compounds may have altered the substrates available for microbial utilization and thereby contributed to this distinct taxonomic profile. However, LEfSe taxa represent group-discriminating features rather than necessarily beneficial or harmful microorganisms. Notably, *Coprocola* was identified as an AOFL-discriminating genus but was negatively correlated with several indices related to intestinal absorption, motility, and antioxidant status. This apparent discordance further indicates that enrichment of an individual taxon cannot be equated with a beneficial functional contribution.

Figure 12 provided an association-based link between microbial variation and host-response domains. *Citrobacter_A*, *Megasphaera_A*, *Ventrisoma*, *CAG-56*, *Clostridium_Q*, and *Coprocola* were negatively correlated with one or more of D-xylose, 5-HT, MTL, and SOD, whereas several of these genera were positively correlated with VIP or MDA. In contrast, *Nanosynococcus*, *UBA946*, and *UMGS1994* were positively associated with 5-HT, SOD, acetate, or propionate. Consistently, MD samples in the RDA were broadly aligned with the VIP and MDA vectors, whereas AOFL samples shifted away from the MD distribution and toward the directions of D-xylose, GAS, MTL, 5-HT, and SOD. These findings support coordinated microbiota–host variation after AOFL intervention. Nevertheless, correlation and RDA do not establish temporal direction or mediation, and PICRUSt2 predictions cannot replace direct functional evidence from metagenomic, metatranscriptomic, or metabolomic analyses (Douglas et al., 2020).

### 4.5 Changes in SCFAs associated with *A. oryzae*-fermented MMF intervention

Acetate, propionate, and butyrate are major microbial SCFAs but differ in their microbial sources, host metabolic fates, and biological functions (Koh et al., 2016; Portincasa et al., 2022; Liu et al., 2024). They should therefore be interpreted separately rather than as a uniform SCFA response. In this study, all three measured SCFAs were significantly lower in the MD group than in the NC group. Acetate and propionate remained lower than NC after UFL intervention. In the AOFL group, acetate was numerically higher than in the MD group, and propionate was numerically higher than in both the MD and UFL groups; however, neither difference reached statistical significance. Butyrate remained below the NC level and showed no corresponding numerical recovery. Thus, the findings do not demonstrate significant restoration of colonic SCFA concentrations by AOFL but instead indicate compound-specific numerical patterns.

The absence of a parallel butyrate response may reflect differences in SCFA-producing microorganisms, substrate utilization, microbial cross-feeding, epithelial uptake, and host metabolism (Peng et al., 2007; Fu et al., 2019; Hodgkinson et al., 2023). Associations between selected genera and acetate or propionate in Figure 12 provide additional ecological context, but they do not establish that either SCFA mediated the host responses.

### 4.6 Limitations and future perspectives

This study has several limitations. First, although AOFL fermentation was standardized by inoculum amount, temperature, duration, and macroscopic mycelial coverage, quantitative endpoint parameters such as pH, residual moisture, fungal biomass, and enzyme activity were not measured. Second, the compounds detected by LC–MS-based untargeted analysis remain candidate annotations because retention times and MS/MS fragment ions were not confirmed using authentic standards. Third, 16S rRNA sequencing and PICRUSt2 only reflect changes in microbial composition and predicted functions, and they cannot replace direct evidence from metagenomics, metatranscriptomics, or metabolomics. This study also does not verify the causal role of microbial changes through antibiotic depletion, fecal microbiota transplantation, specific microbial supplementation, or metabolite intervention experiments. Fourth, this study detects only acetate, propionate, and butyrate, without covering other SCFAs or analyzing butyrate-producing bacteria, SCFA transporters, related receptors, or indices of colonic epithelial energy metabolism. Finally, direct functional endpoints of constipation, including time to first black stool, 6-h fecal pellet output, gastric emptying, small-intestinal propulsion, and colonic transit, were not assessed. The original design prioritized the use of the same animals for terminal collection of colonic contents for SCFA quantification and 16S rRNA sequencing and did not include a separate gastrointestinal-motility validation cohort. Measurement of time to first black stool would have required prior administration of a gastrointestinal tracer, whereas gastric-emptying and small-intestinal-propulsion assays would additionally have required specific fasting and terminal sampling procedures. These procedures might have altered the luminal sampling conditions or introduced non-study tracer materials into the gastrointestinal tract, potentially affecting subsequent SCFA quantification and 16S rRNA sequencing using samples from the same animals. Nevertheless, 6-h fecal pellet output is a nonterminal endpoint, and its omission represents a limitation of the original experimental design. Future studies should prospectively record defecation-related endpoints and use dedicated animal cohorts for marker-based gastrointestinal-transit measurements. Future studies should integrate confirmation with authentic standards, targeted metabolomics, metagenomic sequencing, quantification of key genera, gastrointestinal motility assays, and barrier function validation to further clarify the chemical basis, microbial involvement, and host functional changes underlying the effects of *A. oryzae*-fermented MMF on the spleen-deficiency constipation model.

## 5 Conclusion

This study evaluated the effects of *A. oryzae* fermentation on the putatively annotated chemical composition of MMF and the gut microbiota in mice with spleen-deficiency constipation. Fermentation changed the relative profile of representative putatively annotated compounds, and AOFL was associated with more pronounced changes in intestinal absorptive-function indices, selected motility-related serum markers, hepatic oxidative-stress indices, colonic mucosal structure, and gut microbial composition than UFL. Acetate and propionate were numerically higher in the AOFL group, but the differences were not statistically significant, and butyrate did not show a corresponding recovery. These findings suggest that *A. oryzae* fermentation modifies the putatively annotated chemical composition of MMF and is associated with distinct gut microbial and host-response patterns. However, direct effects on defecation and gastrointestinal transit remain to be established using dedicated functional assays, and statistically significant effects on fecal water content and colonic SCFA concentrations were not demonstrated in the present study. Targeted metabolomics and causality-oriented microbiota experiments are also needed for mechanism validation.

## Data Availability

The 16S rRNA sequencing reads generated in this study have been deposited in the NCBI Sequence Read Archive under BioProject accession number PRJNA1456319.
